# Analysis of the Long-Lived Responses Induced by Immunostimulants and Their Effects on a Viral Infection in Zebrafish (*Danio rerio*)

**DOI:** 10.3389/fimmu.2018.01575

**Published:** 2018-07-09

**Authors:** Margarita Álvarez-Rodríguez, Patricia Pereiro, Felipe E. Reyes-López, Lluis Tort, Antonio Figueras, Beatriz Novoa

**Affiliations:** ^1^Institute of Marine Research (IIM), National Research Council (CSIC), Vigo, Spain; ^2^Department of Cell Biology, Physiology and Immunology, Universidad Autónoma de Barcelona, Bellaterra, Spain

**Keywords:** zebrafish, immunostimulants, β-glucans, stress, tolerance, IFN-γ, kynurenine, TDO

## Abstract

In recent years, the innate immune response has gained importance since evidence indicates that after an adequate priming protocol, it is possible to obtain some prolonged and enhanced immune responses. Nevertheless, several factors, such as the timing and method of administration of the immunostimulants, must be carefully considered. An inappropriate protocol can transform the treatments into a double-edged sword for the teleost immune system, resulting in a stressful and immunosuppressive state. In this work, we analyzed the long-term effects of different stimuli (β-glucans, lipopolysaccharide, and polyinosinic:polycytidylic acid) on the transcriptome modulation induced by Spring Viremia Carp Virus (SVCV) in adult zebrafish (*Danio rerio*) and on the mortality caused by this infection. At 35 days post-immunostimulation, the transcriptome was found to be highly altered compared to that of the control fish, and these stimuli also conditioned the response to SVCV challenge, especially in the case of β-glucans. No protection against SVCV was found with any of the stimuli, and non-significant higher mortalities were even observed, especially with β-glucans. However, in the short term (pre-stimulation with β-glucan and infection after 7 days), slight protection was observed after infection. The transcriptome response in the zebrafish kidney at 35 days posttreatment with β-glucans revealed a significant response associated with stress and immunosuppression. The identification of genes that were differentially expressed before and after the infection seemed to indicate a high energy cost of the immunostimulation that was prolonged over time and could explain the lack of protection against SVCV. Differential responses to stress and alterations in lipid metabolism, the tryptophan–kynurenine pathway, and interferon-gamma signaling seem to be some of the mechanisms involved in this response, which represents the end of trained immunity and the beginning of a stressful state characterized by immunosuppression.

## Introduction

Studies on the effect of immunostimulants in fish aquaculture have been conducted in past decades (1). These immunostimulants include different pathogen-associated molecular patterns (PAMPs), such as polyinosinic:polycytidylic acid [poly(I:C)], lipopolysaccharide (LPS), and β-glucans (2). PAMPs are recognized by host cells through specific pattern-recognition receptors (PRRs), which are germline-encoded host sensors with a key role in innate immunity (3). These PRRs can be proteins expressed mainly in the cell membrane or endosomes of innate immune cells, such as toll-like receptors, cytoplasmic receptors, such as RIG-I-like receptors or NOD-like receptors, or secreted receptors, such as mannose-binding lectin (MBL) of the complement system (3, 4). Nevertheless, the PRRs that sense LPS and β-glucans have not been completely identified in teleosts (5, 6).

Many PRRs can share several signaling pathways, and they have the ability to influence the type, intensity, and duration of the immune response (7). Signaling through PRRs after a first immune stimulus may result in cell reprogramming, which changes and conditions the immune response triggered by a second stimulus. This reprogramming is a host adaptation that coordinates multiple receptors and reflects the plasticity of innate immunity (8).

The term “trained immunity” was coined by Netea et al. in 2011 (9) to refer to cross-protection between infections that occurs independently of T and B cells or to the enhanced non-specific innate immune protection elicited by microbial components or PAMPs. This innate memory is mainly provided by NK cells and macrophages. It has been shown that the priming (or training) of mice with several PAMPs, such as β-glucans, peptidoglycans, or flagellin, can protect against a subsequent lethal infection (10). In contrast, LPS-induced tolerance has been described as the transformation of macrophages to a state that is much less responsive to subsequent challenges with LPS. Such a response should protect organisms from hyperinflammation or sepsis (11). Nevertheless, this suppression can become pernicious when the organism has to fight against a pathogen. Both tolerance and training involve long-term epigenetic reprogramming of the innate immune system after the first contact with a particular pathogen or PAMP (12–14).

It has been demonstrated that β-glucans, which are probably the most commonly used immunostimulant in fish aquaculture, are able to induce metabolic changes that reflect a shift from oxidative phosphorylation to aerobic glycolysis (Warburg effect) during the establishment of trained immunity (15). Because increasing evidence indicates a close relation between metabolism and epigenetic reprogramming (16, 17), the effect of β-glucans on metabolism could mediate changes in the epigenetic pattern. Other metabolic pathways that are independent of glucose metabolism can play a role in the induction of trained immunity by β-glucans (18). Although the identity of the PRR responsible for recognizing β-glucans in fish remains unknown (19), mammals sense this PAMP *via* dectin-1, a C-type lectin receptor that is absent in fish genomes (20). In mammals, training immunity occurs in a dectin-1-dependent manner in macrophages (21).

The outcome of β-glucan stimulation depends on several factors, such as the administration route and fish species, and its protective effect depends on the infectious agent (19). It is also important to keep in mind that only a very small fraction of orally administered β-glucans pass through the intestine to the circulation (22). Therefore, the intraperitoneal administration of β-glucans can provide higher levels of protection, even after a single injection (19). There are many works based on studying the effect of β-glucan pretreatment on the resolution of bacterial infections in fish after either oral or intraperitoneal administration (23–27). However, investigations of resistance against viral infections after β-glucan stimulation in teleosts (28–32), or in vertebrates in general (33–35), are scarce.

The main premise that must be taken into account when considering a substance as a good immunostimulant is the absence of pathogenic, toxic, or other undesirable effects after administration to aquatic organisms. Nevertheless, the administration of a certain immunostimulant can be a stressful stimulus and produce indirect pernicious effects at the immune level. In non-mammalian vertebrates, such as fish, the relation between stress and the immune system is more evident. Neuropeptides and cytokines play roles in the immune and neuroendocrine systems and belong to the same family of molecules (36, 37). A prompt but intense stimulus can result in acute stress; therefore, long-lasting effects on several stress- and immune-related parameters could be observed. Because the head kidney of fish is a unique structure in vertebrates that performs important functions for the endocrine, nervous, and the immune systems (36), it is an ideal tissue for studying the potential effects of immunostimulants.

In this work, we analyzed the long-lived response induced by three PAMPs [LPS, poly(I:C), and β-glucans] in zebrafish at 35 days posttreatment and investigated their effects on survival after Spring Viremia Carp Virus (SVCV) infection. None of the treatments induced protection against viral challenge, and non-significant higher mortalities were even found in the PAMP-treated groups. Kidney samples were collected from non-challenged and SVCV-challenged fish after 24 h, and microarray analyses were conducted. Special attention was paid to β-glucans, which induced a stronger and more specific transcriptome modulation in both uninfected and infected zebrafish. The transcriptome response at 35 days posttreatment with β-glucans revealed interesting changes that could be related to the stress response, immunosuppression, or tolerance. This response to β-glucans seems to be mediated by the interplay among different metabolic and immune processes, such as the alteration of lipid metabolism, the tryptophan–kynurenine pathway and interferon-gamma signaling.

## Materials and Methods

### Fish, Viruses, and Bacteria

Zebrafish were obtained from our experimental facility, where the fish were cultured using established protocols (38, 39) (see http://zfin.org/zf_info/zfbook/zfbk.html). In this work, adult wild-type zebrafish were used for transcriptome and mortality analyses. Double-transgenic *Tg(mpeg:mCherry/mpx:GFP)* zebrafish embryos, in which macrophages are labeled red and neutrophils are labeled green, and wild-type embryos were used in this work for microinjection experiments. Zebrafish were euthanized using a tricaine methanesulfonate (MS-222) overdose (500 mg/l^−1^). For microinjection experiments, larvae were anesthetized by adding two drops of a 0.05% MS-222 solution to a Petri plate with 10 ml of water. Fish care and the challenge experiments were conducted according to the guidelines of the CSIC National Committee on Bioethics under approval number ES360570202001/16/FUN01/PAT.05/tipoE/BNG.

Spring Viremia Carp Virus isolate 56/70 was propagated on epithelioma papulosum cyprini (EPC) carp cells (ATCC CRL-2872) containing MEM (Gibco) supplemented with 2% FBS (Gibco) and 100 µg/ml Primocin (InvivoGen) and titrated in 96-well plates. The TCID_50_/ml was calculated according to the Reed and Muench method (40).

### Plasmid Construction

The zebrafish *interferon gamma 1-2* (*ifng1-2*) gene was amplified by PCR (primers presented in Table 1 in Data Sheet S1 in Supplementary Material), and the PCR product was cloned using the pcDNA 3.1/V5-His TOPO TA Expression Kit (Invitrogen). One Shot TOP10F′ competent cells (Invitrogen) were transformed to generate the plasmid construct (pcDNA 3.1-*ifng1-2*). Plasmid purifications were conducted using the PureLink HiPure Plasmid Midiprep Kit (Invitrogen) following the manufacturer’s instructions.

### Reagents

Lipopolysaccharide from *Escherichia coli* O111:B4, poly(I:C), a TDO-inhibitor [C80C91 or 6-fluoro-3-((1E)-2-(3-pyridinyl)ethenyl)-1H-indole], and an IDO-inhibitor (1-MT or 1-methyl-d-tryptophan) were purchased from Sigma-Aldrich (reference numbers L2630, P1530, SML0287, and 452483, respectively). β-glucans extracted from *Saccharomyces cerevisiae* were obtained from Biotec-Mackzymal. The zebrafish IFN-gamma 1-2 recombinant protein was acquired from Kingfisher Biotech (reference number RP1045Z).

### Stimulation With PAMPs and Challenge With SVCV

Four groups composed of 92 adult zebrafish each were intraperitoneally (i.p.) injected with 20 µl of one of the following treatments: β-glucans (1 mg/ml), LPS (0.75 mg/ml), poly(I:C) (1 mg/ml), and control treatment with phosphate-buffered saline (PBS). After 35 days, half of the individuals were i.p. infected with 20 µl of a SVCV suspension (3 × 10^2^ TCID_50_/ml), and the remaining fish, which served as uninfected controls, were inoculated with viral medium (MEM + 2% FBS + Primocin). For microarray hybridization, a total of 16 fish from each treatment (β-glucans-control, LPS-control, poly(I:C)-control, β-glucans-SVCV, LPS-SVCV and poly(I:C)-SVCV) were sacrificed at 24 h postinfection, and the kidney was removed, yielding four pooled biological replicates (four fish/replicate). Total RNA was extracted as described below, and RNA quality was assessed with the Agilent 2100 Bioanalyzer (RIN ≥ 7).

To determine the long-term effects of the different PAMPs on mortality after challenge with SVCV, the remaining 30 fish from each group were divided into 3 replicates (10 fish per replicate), and mortality was recorded for a period of 3 weeks.

To analyze potential short-term priming with β-glucans, a total of 48 zebrafish were i.p. inoculated with 20 µl of β-glucans (1 mg/ml) or PBS. After a rest period of 7 days, half of the fish were i.p. infected with 20 µl of an SVCV suspension (1.5 × 10^2^ TCID_50_/ml), and the remaining individuals were inoculated with viral medium (control fish). The fish from each group were distributed in three biological replicates (eight individuals/replicate). Mortality was recorded for a period of 3 weeks. In parallel, the same experiment was conducted to analyze gene expression by qPCR at 24 h postinfection in kidney samples (five individual fish by treatment).

### RNA Isolation and cDNA Transcription

Total RNA was isolated using the Maxwell 16 LEV Simply RNA Tissue Kit (Promega) according to the manufacturer’s instructions. cDNA synthesis was conducted with an NZY First-Strand cDNA Synthesis kit (NZYTech) using 0.3 µg of total RNA.

### Microarray Analysis

The 4 × 44K Zebrafish Gene Expression Microarray (V3, AMADID 026437) from Agilent Technologies (Madrid, Spain) was used to analyze gene expression in the different samples (Glucans, LPS, poly(I:C), Glucans-SVCV, LPS-SVCV, and poly(I:C)-SVCV).

The labeling of 1 µg of RNA and hybridizations were carried out at the Universidad Autónoma de Barcelona microarray facility, in compliance with the Minimum Information about a Microarray Experiment (MIAME) standards (41). The signal was captured, processed, and segmented using an Agilent G2565B scanner (Agilent Technologies, Madrid, Spain) with Agilent Feature Extraction Software (v9.5) protocol GE1-v5_95 using an extended dynamic range and preprocessing by Agilent Feature Extraction v9.5.5.1.

The results for the fluorescence intensity data and quality annotations were imported into GeneSpring GX version 14.9 (Agilent Technologies). All of the control features were excluded from the subsequent analyses. After grouping the biological replicates (four replicates per treatment), entities with an expression level between the 20th and 95th percentiles in the raw data were retained and used in the subsequent analyses. The gene-level experiment was carried out by normalizing the data *via* a percentile shift at the 75th percentile and using the median of all samples as the baseline transformation. Differentially expressed genes were identified through volcano plot filtering. An unpaired *t*-test was conducted without correction, and data were considered significant at *p* ≤ 0.01. The fold-change cut-off was set at 1.5. Raw and normalized data were deposited in the NCBI’s Gene Expression Omnibus (GEO, http://www.ncbi.nlm.nih.gov/geo/) and are available under the accession number GSE113241. The heatmaps included in this work were also constructed with GeneSpring GX version 14.9.

### Microarray Validation and qPCR

Microarray results were validated through qPCR by analyzing the expression of nine different genes in the same samples used to hybridize the microarray, specifically, in the samples Glucans-SVCV and PBS-SVCV. Those genes were *nk-lysin a* (*nkla*), *nk-lysin d* (*nkld*), *interferon-gamma 1-2* (*ifng1-2*), *tryptophan 2,3-dioxygenase a* (*tdo2a*), *cyclin y* (*ccny*), *membrane-bound O-acyltransferase domain containing 4* (*mboat4*), *pyruvate dehydrogenase kinase isozyme 3a* (*pdk3a*), *nuclear receptor subfamily 1 group D member 2a* (*nr1d2a*), and *adrenomedullin 2a* (*adm2a*). The correlation between microarray results and qPCR fold-change values was analyzed using the Pearson correlation coefficient. Specific qPCR primers were designed using the Primer3 software (42), and their amplification efficiency was calculated using seven serial twofold dilutions of cDNA with the threshold cycle (CT) slope method (43). Primer sequences are listed in Table 1 in Data Sheet S1 in Supplementary Material. Individual qPCR reactions were carried out in a 25 µl reaction volume using 12.5 µl of SYBR GREEN PCR Master Mix (Applied Biosystems), 10.5 µl of ultrapure water (Sigma-Aldrich), 0.5 µl of each specific primer (10 µM), and 1 µl of fivefold diluted cDNA template in MicroAmp optical 96-well reaction plates (Applied Biosystems). All reactions were performed using technical triplicates in a 7300 Real-Time PCR System thermocycler (Applied Biosystems), with an initial denaturation (95°C, 10 min) followed by 40 cycles of a denaturation step (95°C, 15 s) and one hybridization-elongation step (60°C, 1 min). The relative expression levels of the genes were normalized using *18S ribosomal RNA* (*18* *s*) as a reference gene following the Pfaffl method (43).

### Panther and Blast2GO Transcriptome Analysis

Lists of genes that were significantly up- and downregulated relative to the corresponding control were imported into the Protein ANalysis THrough Evolutionary Relationships (PANTHER) Classification System Version 13.0 (44) to classify the modulated genes into Gene Ontology (GO)-Slim Biological Processes.

Enrichment analysis was carried out using the Blast2GO software (45, 46) to detect the major GO terms that were overrepresented in relation to the entire microarray chip (*p*-values ≤0.01 for biological process and ≤0.005 for molecular function).

### Overexpression of *ifng1-2* in Zebrafish Larvae

The expression plasmid pcDNA3.1-*ifng1-2* and the corresponding control plasmid (pcDNA3.1) were microinjected into one-cell stage zebrafish embryos using a glass microneedle incorporated into the Narishige MN-151 micromanipulator and the Narishige IM-30 microinjector. Wild-type zebrafish embryos were microinjected with 200 pg/egg (final volume of 2 nl, diluted in PBS) of the recombinant plasmid or the empty plasmid (60 embryos per treatment). Three days after plasmid injection [3 days postfertilization (dpf)], half of the larvae from each treatment were infected through microinjection into the duct of Cuvier (47) with 2 nl of an SVCV suspension (1.5 × 10^5^ TCID_50_/ml) diluted in PBS and 0.1% phenol red. The remaining larvae were microinjected with the same volume of PBS + 0.1% phenol red (uninfected controls). The infections were carried out at 23°C. Larvae from each group (pcDNA3.1-Control, pcDNA3.1-*ifng1-2-*Control, pcDNA3.1-SVCV, and pcDNA3.1-*ifng1-2*–SVCV) were distributed in three biological replicates (10 larvae/replicate) into 6-well plates. Mortality was assessed during the next 5 days postinfection (dpi). This experiment was replicated three times. Samples were also collected to assess by qPCR the correct replication of the expression plasmid pcDNA3.1-*ifng1-2* in non-infected larvae (3 biological replicates, 10 larvae/replicate).

To analyze the effect of *ifng1-2* overexpression in macrophages and neutrophils, double-transgenic *Tg(mpeg:mCherry/mpx:GFP)* embryos were also microinjected with 200 pg/egg of pcDNA3.1-*ifng1-2* and pcDNA3. At 3 dpf, half of the larvae from each treatment were challenged with SVCV (10^3^ TCID_50_/ml). The remaining larvae were microinjected with the same volume of PBS + 0.1% phenol red (uninfected controls). The cell morphology was analyzed in 4 dpf larvae using a TSC SPE confocal microscope (Leica), and images were processed with the LAS AF software (Leica).

### Effect of Ifng1-2 and TDO and IDO Inhibitors on Mortality After an SVCV Challenge in Adult Zebrafish

Six groups of 24 adult zebrafish were i.p. inoculated with a 10 µl volume of one of the following treatments: recombinant zebrafish Ifng1-2 (0.003 µg/µl) co-administered with SVCV, a TDO inhibitor (680C91; 1.5 µg/µl) in combination with an IDO inhibitor (1-MT; 1.5 µg/µl) and SVCV, SVCV alone, Ifng1-2 without SVCV, a TDO-inhibitor and an IDO-inhibitor without SVCV, or viral medium alone (MEM + 2% FBS + Primocin + 0.08% DMSO). The SVCV concentration was 3 × 10^2^ TCID_50_/ml, and all treatments were diluted in viral medium. The fish from each group were distributed in three biological replicates (eight individuals/replicate). Mortality was recorded during the next 3 weeks.

### Statistical Analyses

For the survival experiments, Kaplan–Meier survival curves were constructed and analyzed with the log-rank (Mantel–Cox) test. The Pearson correlation coefficient was calculated using the SPSS software version 23.0.

## Results

### Effect of Different PAMPs on the Zebrafish Transcriptome After 35 Days

**Figure 1:**
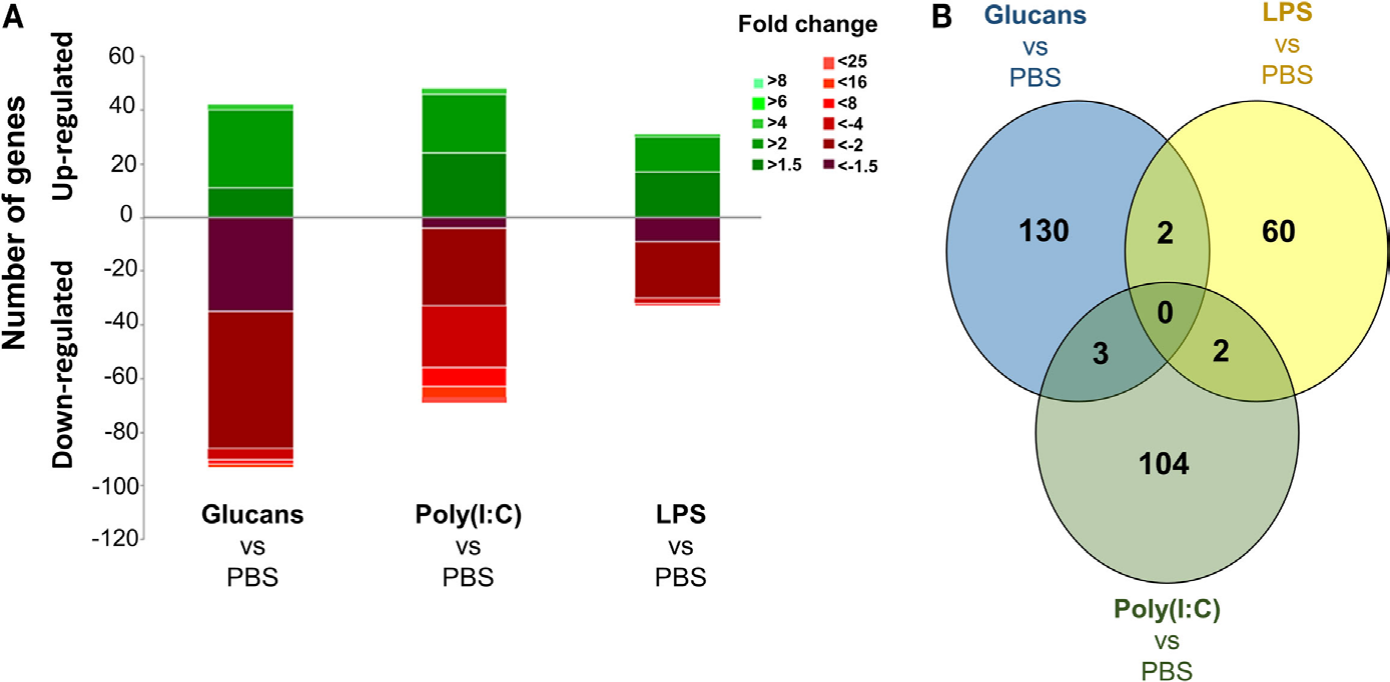
Analysis of gene modulation in the zebrafish kidney at 35 days poststimulation with the three pathogen-associated molecular patterns (PAMPs) compared to individuals inoculated with phosphate-buffered saline. **(A)** Stacked column chart reflecting the distribution of the DEGs. Statistically significant differentially expressed genes are subdivided according to intensity (fold change) and sense (up- and downregulation). **(B)** Venn diagram showing the number of shared and exclusive modulated genes in the three treated groups. Even after 35 days, the response was found to be very exclusive depending on the PAMP.

The number and intensity of modulated genes in the zebrafish kidney 35 days after the administration of three different PAMPs are shown as stacked column charts in Figure 1A. Additionally, the genes that were significantly affected by the long-term administration of β-glucans, poly(I:C), and LPS and their corresponding fold-changes are represented in Data Sheet S2 in Supplementary Material.

The number of differentially expressed genes (DEGs) in the PAMP-treated fish compared to the control (PBS-injected) fish indicates that the effect of the immunostimulation endured over time. The response to LPS administration was the most equilibrated, with a lower number of DEGs, 64 genes in total, and similar quantities of up- and downregulated genes, 31 and 33 genes, respectively. Stimulation with β-glucans and poly(I:C) induced similar responses in terms of the number of modulated genes, with 135 and 109 DEGs, respectively, and a predominance of downregulated genes was observed (Figure 1A). Nevertheless, a Venn diagram comparing the number of shared and exclusive DEGs among the three treatments revealed strikingly different gene modulation patterns (Figure 1B). No DEGs were common to the three PAMPs. In addition, only two genes were shared by glucans and LPS or LPS and poly(I:C), and three DEGs were common to glucans and poly(I:C). These results reflect an exclusive reprograming of the transcriptome for each PAMP.

### Differential Responses to SVCV Challenge After Previous Administration of Different PAMPs and Effects on Survival

**Figure 2:**
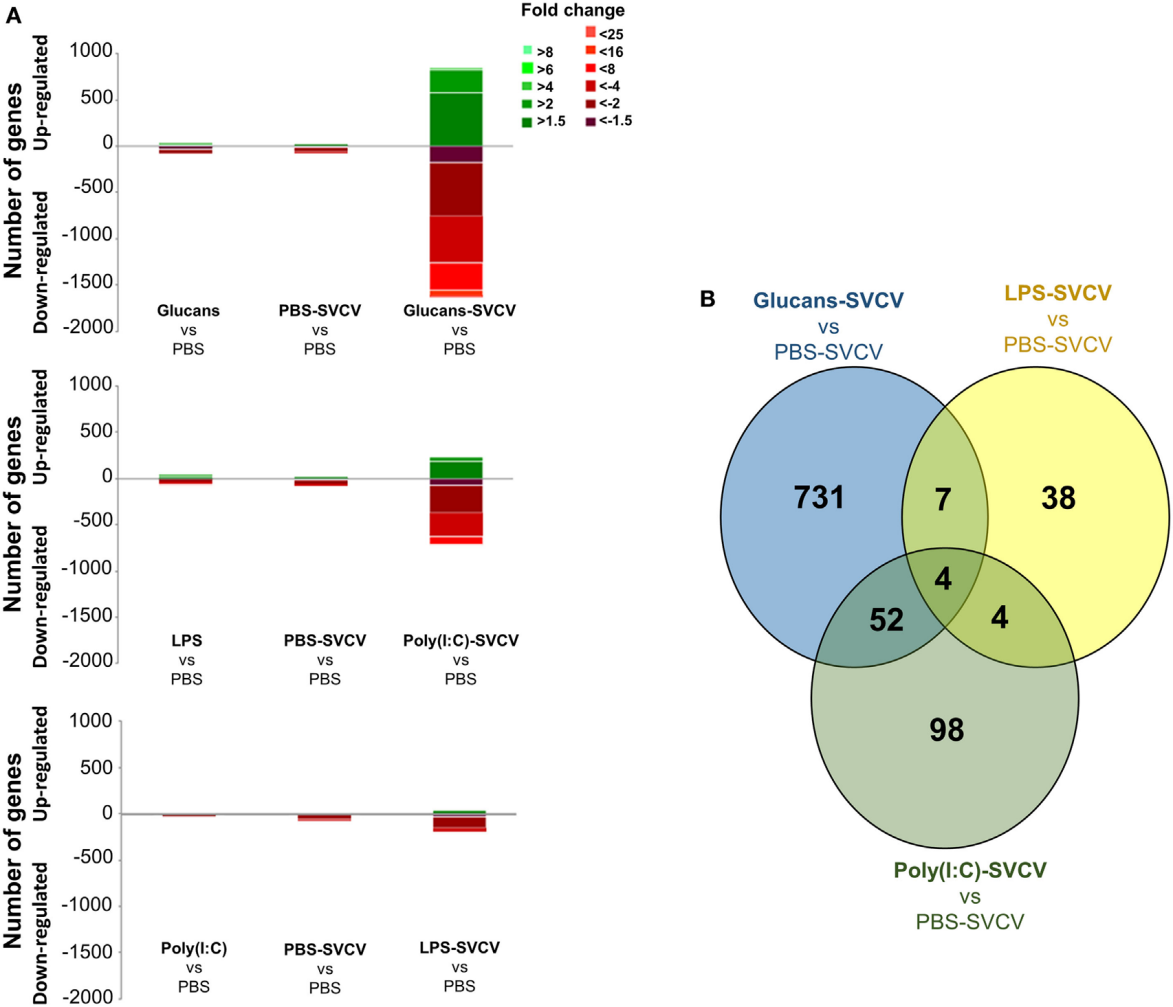
Analysis of gene modulation in the zebrafish kidney at 24 h postinfection with Spring Viremia Carp Virus (SVCV) in individuals that were previously stimulated with pathogen-associated molecular patterns (PAMPs). **(A)** Stacked column chart reflecting the distribution of the DEGs compared to individuals inoculated with phosphate-buffered saline (PBS). Statistically significant differentially expressed genes are subdivided according to intensity (fold change) and sense (up- and downregulation). **(B)** Venn diagram showing the number of shared and exclusive modulated genes after SVCV challenge in the three treated groups compared to PBS-SVCV fish. As observed in the absence of infection, the response was very exclusive depending on the PAMP.

When the transcriptome response to SVCV was analyzed at 24 h postinfection in fish that were previously stimulated with PAMPs or not (Data Sheet S3 in Supplementary Material), the number of DEGs relative to PBS-inoculated zebrafish was found to be higher when PAMP stimulation and viral infection were combined than when the fish received PAMP stimulation or SVCV infection alone (Figure 2A). This multiplied response was drastic in the case of the individuals pretreated with β-glucans and then infected with SVCV after 35 days. In these individuals (Glucans-SVCV), the number of DEGs relative to the PBS group was 2,486, whereas only 135 and 113 genes were modulated in the Glucans and PBS-SVCV groups, respectively. This difference implies that the response to the virus was 21 times higher in the Glucans-SVCV group than in the PBS-SVCV group and 18 times higher in the Glucan-SVCV group than in the Glucans group. The additive effect of the two stimuli was much lower in the case of poly(I:C) [946 DEGs in poly(I:C)-SVCV] and especially LPS (250 DEGs in LPS-SVCV) (Figure 2A).

When the transcriptome response to SVCV challenge in fish that were previously treated with different stimuli was compared with the response to infection alone (PBS-SVCV group) (Data Sheet S4 in Supplementary Material) through a Venn diagram, the number of exclusive DEGs was also high, representing 92.1% in the Glucans-SVCV group, 62% in the poly(I:C)-SVCV group, and 71.7% in the LPS-SVCV group (Figure 2B).

**Figure 3:**
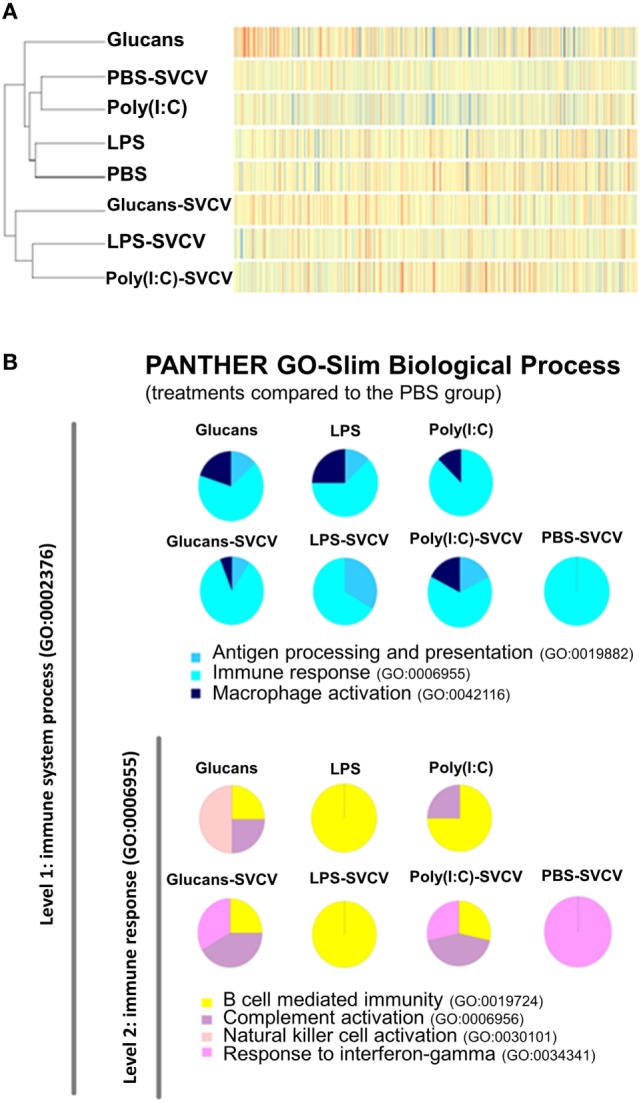
Similarity analysis of the transcriptome response at 35 days post-immunostimulation in the presence or absence of Spring Viremia Carp Virus (SVCV) infection. **(A)** Dendrogram and heatmap representing the overall microarray results. The samples were divided into two clusters, one containing the pathogen-associated molecular patterns (PAMP)-stimulated groups and the individuals infected in the absence of pre-stimulation, and the other including the groups that were pre-stimulated with PAMPs and then infected with SVCV. In both clusters, the β-glucans formed a separate branch, indicating that this stimulus induced the most differential response. **(B)** Functional classification of DEGs in the zebrafish kidney between the different treatments and the phosphate-buffered saline-treated group according to Slim Biological Process Gene Ontology Terms. The level 1 category “Immune System Process” was selected, and within that category, the level 2 category “Immune Response.” After SVCV challenge, the gene categorization was conditioned by previous stimulation with a PAMP.

A dendrogram and a heatmap of the overall microarray data revealed two main clusters of similarity (Figure 3A). In the first cluster, zebrafish groups that were inoculated with PAMPs or PBS but uninfected were placed together. Interestingly, in this cluster, the PBS-SVCV group was also included, which was closely related to the poly(I:C)-injected fish. The expression pattern of the Glucans group was the most different within this cluster and formed an independent branch. Therefore, the treatment with β-glucans induced a stronger long-lasting effect on the transcriptome. In the other cluster, the groups that were stimulated with the three PAMPs and then infected with SVCV were included, with the Glucans-SVCV group being the most different.

The DEGs in all treatment groups, infected and uninfected, relative to the PBS uninfected group, were categorized according to their biological processes using the PANTHER software. In the “Immune response” category, the three PAMP-treated groups presented genes in “B cell mediated immunity”; Glucans and poly(I:C) also presented genes in “complement activation,” whereas only Glucans presented genes belonging to the “natural killer cell activation” category (Figure 3B). After infection in the absence of previous immunostimulation (PBS-SVCV), the response seemed to be dominated by “response to interferon-gamma,” but individuals that were previously treated with PAMPs maintained certain mechanisms triggered by the PAMP even after SVCV infection (Figure 3B), presenting a completely different response than the non-immunostimulated group. This finding could indicate a longer conditioning effect after the first stimulus (PAMP) than after the second stimulus (SVCV).

**Figure 4:**
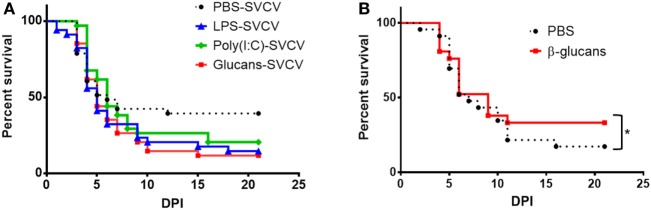
Effect of pathogen-associated molecular patterns (PAMPs) on the survival of adult zebrafish after a Spring Viremia Carp Virus (SVCV) challenge. **(A)** Kaplan–Meier survival curves after an SVCV i.p. challenge. Fish were stimulated with PAMPs or inoculated with phosphate-buffered saline (PBS), and 35 days after a single-dose administration, they were i.p. challenged with SVCV. No significant differences were observed among the different groups, although the PAMP-treated zebrafish showed a lower survival rate. **(B)** Kaplan–Meier survival curves after SVCV i.p. challenge. Fish were previously inoculated with β-glucans or PBS and i.p. challenged with SVCV 7 days after a single-dose administration. A slight but significant increase in the survival rate was observed in fish that were previously treated with β-glucans. Significant differences are represented by asterisks (*p < 0.05).

Despite these amplified responses to the virus in individuals that were previously stimulated with PAMPs, a reduction of mortality was not observed (Figure 4A). In contrast, although the differences were not found to be statistically significant, higher mortality after SVCV infection was observed in the three groups stimulated with PAMPs.

### The Modulated Transcriptome of β-Glucan-Treated Fish at 35 Days Poststimulation Suggests an Immunosupressive State

Due to the larger/stronger response of the individuals treated with β-glucans, we focused our attention on this immunostimulant. Because the SVCV infections conducted in this work were performed after a long resting period (35 days after stimulation with the different PAMPs), we wanted to determine if β-glucans were able to provide protection against SVCV at 7 days poststimulation. In this case, β-glucan administration slightly but significantly increased the survival rate from 17.4 to 33.3% (Figure 4B), indicating that the effect of this stimulus after 7 days benefits the resolution of the disease.

To determine why β-glucans were not able to protect after a long rest period but had a profound effect on gene expression, we analyzed in more detail the modulation of the transcriptome in treated fish 35 days after i.p. injection (Data Sheet S5 in Supplementary Material). When the complete set of DEGs was taken into account, interesting clues about the effects of long-term stimulation with β-glucans were revealed (Table 2 in Data Sheet S1 in Supplementary Material). In this case, it is interesting to highlight the enriched GO terms related to steroid hormones in the comparison Glucans-SVCV vs. Glucans, such as “intracellular steroid hormone receptor signaling pathway” and “response to epinephrine,” which do not appear in the comparison PBS-SVCV vs. PBS. These terms are related to the activation of stress response pathways. Indeed, “response to epinephrine” is already represented in the comparison Glucans vs. PBS. It is also worth noting that terms related to the antiviral responses that were observed after SVCV infection in the comparison PBS-SVCV vs. PBS (such as chemotaxis and inflammation) were not observed after infection in the comparison Glucans-SVCV vs. Glucans (Table 2 in Data Sheet S1 in Supplementary Material).

If we focus on the GO terms associated with the top 25 most up- and downmodulated genes (Table 1) in the β-glucan-treated fish after infection relative to non-stimulated but infected fish (Table 1), it is interesting to note that the GO terms enriched in the upregulated genes were related to lipid transport (“medium-chain fatty acid transport”), the catabolism of amino acids, especially tryptophan (“tryptophan catabolic process to acetyl-CoA,” “tryptophan catabolic process to kynurenine,” “tryptophan 2,3-dioxigenase activity,” “tyrosine metabolic process,” and “l-phenylalanine catabolic process”), and interferon-gamma activity (“interferon-gamma receptor binding”). Interestingly, an immunosuppressive profile seems to be present in individuals that previously received β-glucans, represented by GO categories enriched in the most downmodulated genes that were directly related to pathogen recognition (“detection of diacyl bacterial lipopeptide,” “toll-like receptor 6 signaling pathway,” and “MyD88-dependent toll-like receptor signaling pathway”) and inflammation (“positive regulation of interleukin-6 biosynthetic process,” “T-helper 1 type immune response,” and “regulation of cytokine secretion”) (Table 2).

**Table 1:** Top 25 most up- and downregulated genes in β-glucan-treated zebrafish compared to unstimulated fish in the absence (A) or presence (B) of Spring Viremia Carp Virus (SVCV) infection.

**(A) Top 25-DEGs glucans vs. phosphate-buffered saline (PBS)**	

**Gene name**	**FC**
**Upregulated genes**	
F-box protein 36a	4.1
G protein-coupled receptor 35, tandem duplicate 1	4.0
Crystallin, gamma M2c	3.8
Golgin subfamily A member 6-like protein 22	3.3
Troponin I type 1b	3.2
Fibronectin type III domain-containing protein 4-like	3.1
Tumor necrosis factor receptor superfamily, member 9b	3.0
Phospholipase C-like 1	3.0
Adhesion G protein-coupled receptor F8	2.8
Relaxin/insulin-like family peptide receptor 3.3b	2.8
ERBB receptor feedback inhibitor 1	2.7
Inner membrane protein, mitochondrial (mitofilin)	2.6
Heterogeneous nuclear ribonucleoprotein A3	2.4
PDZ domain containing 3b	2.4
Testis-enhanced gene transcript (BAX inhibitor 1)	2.4
Cerebellin 9	2.4
Sema domain, immunoglobulin domain (Ig), short basic domain, secreted (semaphorin) 3bl	2.3
Protein phosphatase 6, regulatory subunit 2	2.3
Cilia and flagella associated protein 126	2.2
Actin, alpha 1a, skeletal muscle	2.2
FAD-dependent oxidoreductase domain containing 2	2.2
Angiopoietin-like 2b	2.1
Transgelin	2.1
DnaJ (Hsp40) homolog, subfamily C, member 28	1.8
THAP domain containing 5	1.7

**Downregulated genes**	

Cytochrome P450, family 8, subfamily B, polypeptide 3	8.7
Angiotensinogen	6.5
Small integral membrane protein 1	6.1
Growth arrest and DNA-damage-inducible, beta a	4.5
Ephrin-A3b	4.4
Zinc finger protein 1065	4.3
Chemokine (C-X-C motif) receptor 5	4.2
Subcommissural organ spondin	4.0
Dachshund b	3.9
Tubulin tyrosine ligase-like family, member 12	3.8
ATPase, class I, type 8B, member 5a	3.4
Forkhead box F2b	3.4
Potassium voltage-gated channel, Shaw-related subfamily, member 1b	3.3
Proteoglycan 4b	3.3
Zinc finger protein 1059	3.2
Tumor necrosis factor receptor superfamily, member 21	3.1
Growth arrest and DNA-damage-inducible, alpha, b	3.1
CCAAT/enhancer binding protein (C/EBP), delta	3.1
Carboxypeptidase A4	3.0
Elastase 3 like	2.9
Mitogen-activated protein kinase kinase kinase 1	2.9
TBC1 domain family, member 12a	2.8
*Danio rerio* serum/glucocorticoid regulated kinase	2.6
*D. rerio* endothelin 1	2.6
*D. rerio* Smith–Magenis syndrome chromosome region, candidate 8b	2.5

**(B) TOP 25-DEGs glucans-SVCV vs. PBS-SVCV**	

**Upregulated genes**	

Tryptophan 2,3-dioxygenase a	21.8
Interferon, gamma 1-2	6.2
Fatty acid binding protein 1b, liver, tandem duplicate 2	4.8
Cytochrome P450, family 3, subfamily A, polypeptide 65	4.5
Geminin, DNA replication inhibitor	4.4
Microsomal triglyceride transfer protein	4.3
Inositol 1,4,5-trisphosphate receptor-interacting protein	4.2
Heparanase	4.2
Tumor protein p63 regulated 1-like	4.2
Receptor-type tyrosine-protein phosphatase S precursor	3.7
Major facilitator superfamily domain containing 2ab	3.4
RAS (RAD and GEM)-like GTP-binding 1	3.2
Homogentisate 1,2-dioxygenase	3.2
ISG15 ubiquitin-like modifier	3.1
TSC22 domain family, member 3	3.0
UDP glucuronosyltransferase 1 family polypeptide a1	3.0
T-cell activation RhoGTPase activating protein b	3.0
VHSV-inducible protein—*Salmo salar*	2.9
UDP glucuronosyltransferase 1 family, polypeptide A7	2.9
Interferon-induced very large GTPase 1-like	2.8
Mastermind-like transcriptional coactivator 3	2.7
Forkhead box A2	2.7
Complement component bfb	2.7
Cysteine-serine-rich nuclear protein 1a	2.6
Interferon regulatory factor 1b	2.6

**Downregulated genes**	

Leucine-rich repeat and Ig domain containing 4a	45.8
Cyclin Y	29.2
Desumoylating isopeptidase 1a	27.3
GTPase IMAP family member 4	26.5
Leucine-rich repeat, immunoglobulin-like and transmembrane domains 1b	25.4
Gap junction alpha-1 protein-like	23.1
Vomeronasal 2 receptor, c2	22.4
Motilin receptor	20.0
Membrane-bound O-acyltransferase domain containing 4	19.8
Neural cell adhesion molecule 2	17.7
Hyperpolarization activated cyclic nucleotide-gated potassium channel 4	16.2
Transmembrane protein 63B	14.7
*D. rerio* neuropeptide FF receptor 1-like	14.7
Semaphorin 7A, GPI membrane anchor	14.6
Transmembrane protein 237a	14.3
Protein phosphatase 1, regulatory subunit 3Aa	14.2
Toll-like receptor 1	14.1
Ceramide synthase 2-like	13.7
Lysine (K)-specific methyltransferase 2E	13.4
Uridine-cytidine kinase 1	13.3
Parathyroid hormone 1 receptor a	13.2
Zinc finger, MYND-type containing 12	13.2
FEV (ETS oncogene family)	12.9
Pyruvate dehydrogenase kinase, isozyme 3a	12.5
finTRIM family, member 58	12.5

**Table 2:** Gene ontology (GO) enrichment analysis of the top 25 most modulated genes in fish that previously received β-glucans and were then infected [Glucans-Spring Viremia Carp Virus (SVCV)] compared to untreated and infected fish (PBS-SVCV).

GO name	GO category	*p* -Value
**TOP 25 upregulated DEGs GLUCANOS-SVCV vs. PBS-SVCV**		

Medium-chain fatty acid transport	BP	0.001
Central nervous system myelin formation		0.002
Tryptophan catabolic process to acetyl-CoA		0.002
Tyrosine metabolic process		0.002
Positive regulation of transcription of notch receptor target		0.002
Lysophospholipid transport		0.002
Long-chain fatty acid transport		0.004
Transcytosis		0.004
Tryptophan catabolic process to kynurenine		0.004
l-phenylalanine catabolic process		0.005
Negative regulation of DNA replication		0.005
Oligodendrocyte cell fate commitment		0.005
Establishment of blood–brain barrier		0.005
Axial mesoderm morphogenesis		0.009

Homogentisate 1,2-dioxygenase activity	MF	0.001
Interferon-gamma receptor binding		0.002
Tryptophan 2,3-dioxygenase activity		0.002
Lipid transporter activity		0.003

**TOP 25 downregulated DEGs GLUCANOS-SVCV vs. PBS-SVCV**		

Detection of diacyl bacterial lipopeptide	BP	0.001
Positive regulation of interleukin-6 biosynthetic process		0.001
Toll-like receptor 6 signaling pathway		0.001
Peptidyl-serine octanoylation		0.001
T-helper 1 type immune response		0.001
Positive regulation of inositol phosphate biosynthetic process		0.001
Regulation of cytokine secretion		0.002
CTP salvage		0.004
MyD88-dependent toll-like receptor signaling pathway		0.005
UMP salvage		0.006
Regulation of heart rate		0.007
Pyrimidine nucleobase metabolic process		0.009
Dorsal aorta development		0.009

Parathyroid hormone receptor activity	MF	0.002
Peptide hormone binding		0.003
Peptidyl-lysine acetyltransferase activity		0.005
Uridine kinase activity		0.005

*BP, biological process; MF, molecular function*.

### β-Glucans Modify Lipid Metabolism by Inhibiting the Synthesis of Fatty Acids and Cholesterol Before and After Viral Infection

**Figure 5:**
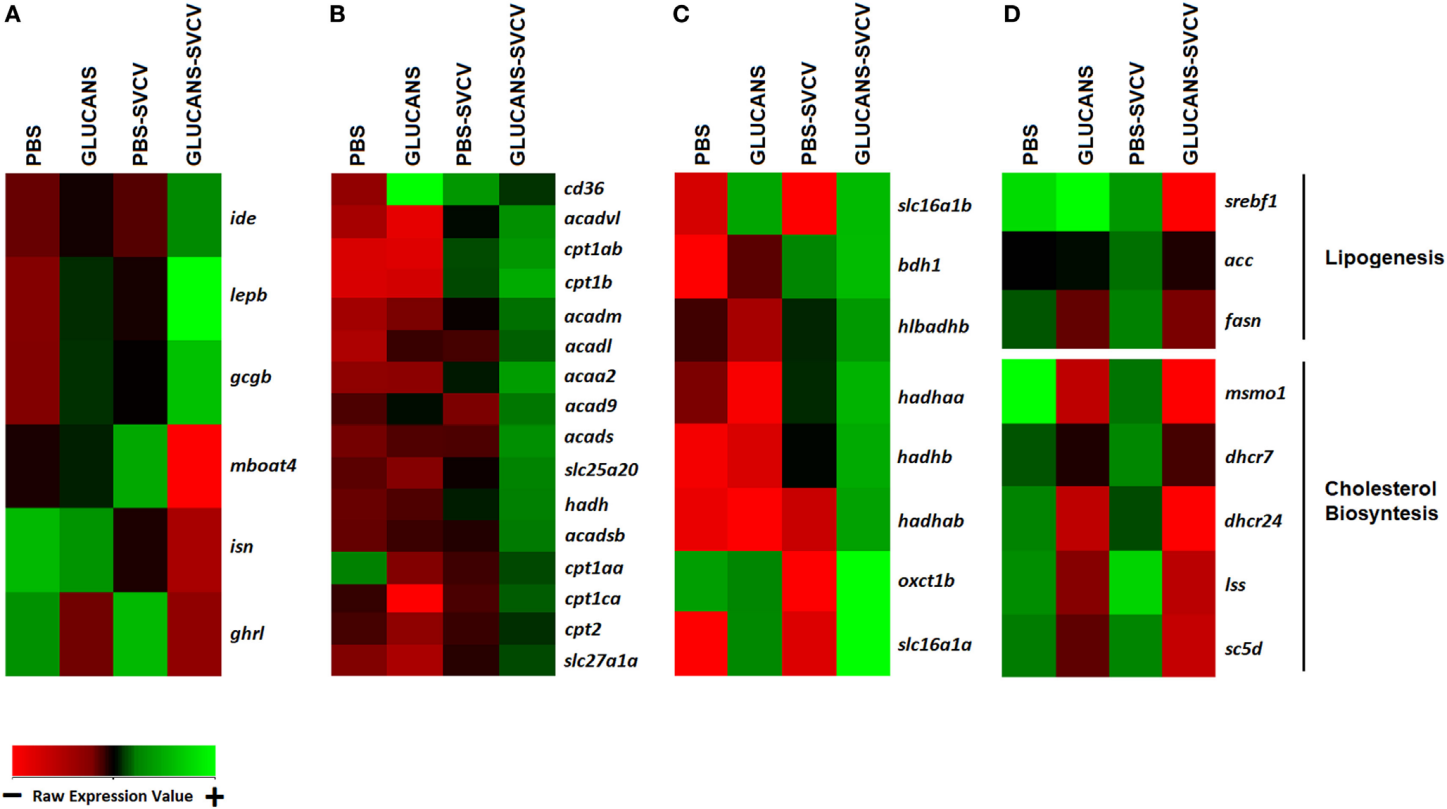
Heatmaps representing different components related to lipid metabolism and transport. **(A)** Hormone genes involved in lipid metabolism. **(B)** Genes encoding key components of fatty acid oxidation. **(C)** Ketolysis. **(D)** Lipogenesis and cholesterol biosynthesis. A color gradient scheme representing gene modulation (red: lower expression; green: higher expression) is shown on the bottom.

Lipid transport seemed to characterize the response to SVCV in individuals that were previously treated with β-glucans. This finding was consistent with the modulation of genes that were overexpressed, such as *fatty acid-binding protein 1b, liver, tandem duplicate 2 (fabp1b.2), microsomal triglyceride transfer protein (mtp), and major facilitator superfamily domain containing 2ab (mfsd2ab)* (Table 1). It is known that β-glucans induce changes in lipid metabolism, and these metabolic changes could condition the immune status of the host. To determine the differential expression profiles of genes encoding key proteins in different lipid metabolic pathways [fatty acid oxidation (FAO), ketolysis, lipogenesis, and cholesterol biosynthesis], specific heatmaps for each pathway were constructed (Figure 5).

Hormone genes that could underlie the modulation of these pathways are shown in Figure 5A. The genes *leptin* (*lepb*), *insulin-degrading enzyme* (*ide*), and *glucagon* (*gcgb*), whose expression is potentiated by double stimulation with β-glucans and SVCV, are inhibitors of the synthesis of lipids and favor lipolysis and ketolysis (48, 49). On the other hand, an opposite effect was observed for *insulin* (*isn*), *ghrelin* (*ghrl*) and *membrane-bound o-acyltransferase domain containing 4* (*mboat4*), which endode proteins that activate lipogenesis (50, 51). In accordance with these observations, the heatmaps associated with lipid metabolism showed that Glucans-SVCV zebrafish present a clear induction of FAO (Figure 5B) and ketolysis (Figure 5C) and a significant inhibition of fatty acid syntesis (Figure 5D), which is reflected in the downregulation of key components of lipid synthesis, such as *sterol regulatory element binding transcription factor 1* (*srebf1*), *fatty acid synthase* (*fasn*), and *acetyl-coA carboxylase (acc)*, and genes of the cholesterol synthesis pathway (Figure 5D). The effect on lipid metabolism was also observed in fish that were stimulated with β-glucans alone, in which the inhibition of cholesterol synthesis, the downregulation of *ghrl*, and the upregulation of the fatty acid translocase *scavenger receptor class B, member 3* (*cd36*), which is essential for fatty acid import into cells and efficient oxidation, were observed.

### Effect of Ifng1-2 on Macrophage Activation and Survival After an SVCV Challenge

The transcriptome of infected fish that were previously treated with β-glucans was enriched in the immune term “interferon-gamma receptor binding,” as a result of the high expression of the *ifng1-2* gene, among others (Table 1). IFN-γ is an important activator of macrophages in mammals, with immunostimulatory and immunomodulatory properties. Nevertheless, in this case, Ifng1-2 seeems to be associated to an immunosuppressive state. For that reason, we wondered if this molecule has a pernicious effect in zebrafish during an infection with SVCV or if its association with the immunosuppression depends on other factors.

**Figure 6:**
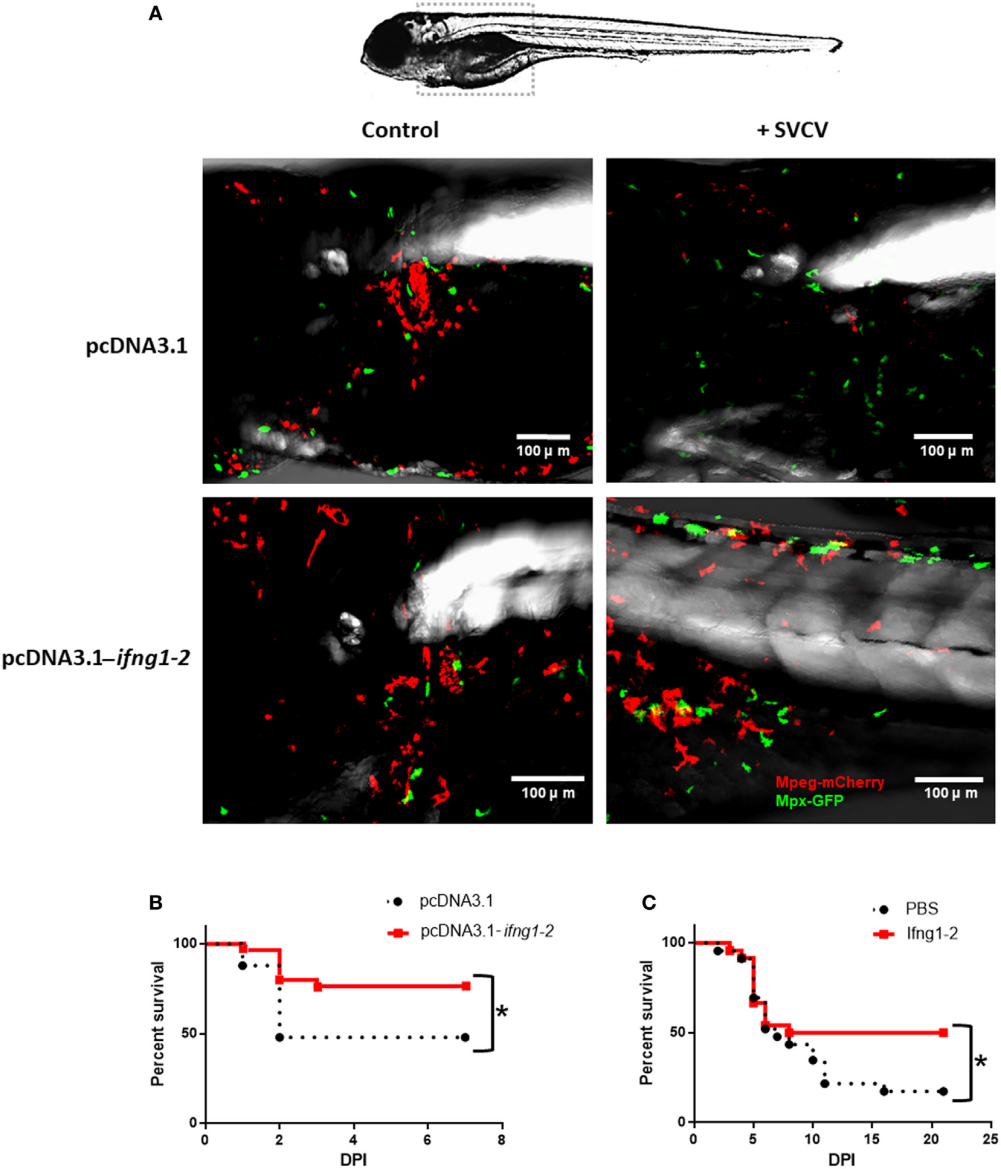
Effect of Ifng1-2 on the activation of immune cells and on survival against Spring Viremia Carp Virus (SVCV). **(A)** Zebrafish larvae overexpressing *ifng1-2* show the activation of macrophages and neutrophils, with an evident change in morphology from a spherical morphology to a dendritic morphology. **(B)** Kaplan–Meier survival curves after SVCV challenge in zebrafish larvae overexpressing or not overexpressing *ifng1-2* **(C)** Kaplan–Meier survival curves after SVCV challenge in adult zebrafish co-inoculated or not co-inoculated with the Ifng1-2 recombinant protein. Significant differences are represented by asterisks (*p < 0.05).

An expression plasmid encoding *ifng1-2 (*pcDNA3.1-*ifng1-2)* was constructed, and its correct replication was confirmed in 3 dpf larvae (Figure 1 in Data Sheet S1 in Supplementary Material). The plasmid pcDNA3.1-*ifng1-2* and the corresponding empty plasmid (pcDNA3.1) were microinjected into double-transgenic *Tg(mpeg:mCherry/mpx:GFP)* zebrafish embryos alone or in combination with SVCV. When we compared the morphology of macrophages and neutrophils in 4 dpf larvae, it was evident that larvae overexpressing *ifng1-2* presented a different morphology than the controls for both cell types (Figure 6A). Both neutrophils and macrophages changed from a spherical morphology to a dendritic morphology, which was especially evident in infected and pcDNA3.1-*ifng1-2-*treated fish; in the case of macrophages, these changes probably reflect the activation of these cells.

The overexpression of *ifng1-2* in wild-type zebrafish larvae also revealed the ability to significantly increase survival after an SVCV challenge (Figure 6B). The survival of the empty plasmid-treated fish was 48%, whereas this rate increased to 76.7% in individuals that were previously inoculated with the expression plasmid pcDNA3.1-*ifng1-2*. This protective capability against SVCV was also tested in adult zebrafish using the recombinant protein (Figure 6C). In this case, the untreated fish exhibited a survival rate of 17.4%, but individuals that were inoculated with Ifng1-2 significantly increased their survival to 50%.

### The Alteration of the Tryptophan–Kynurenine Pathway Influences the Survival Rate After SVCV Infection

**Figure 7:**
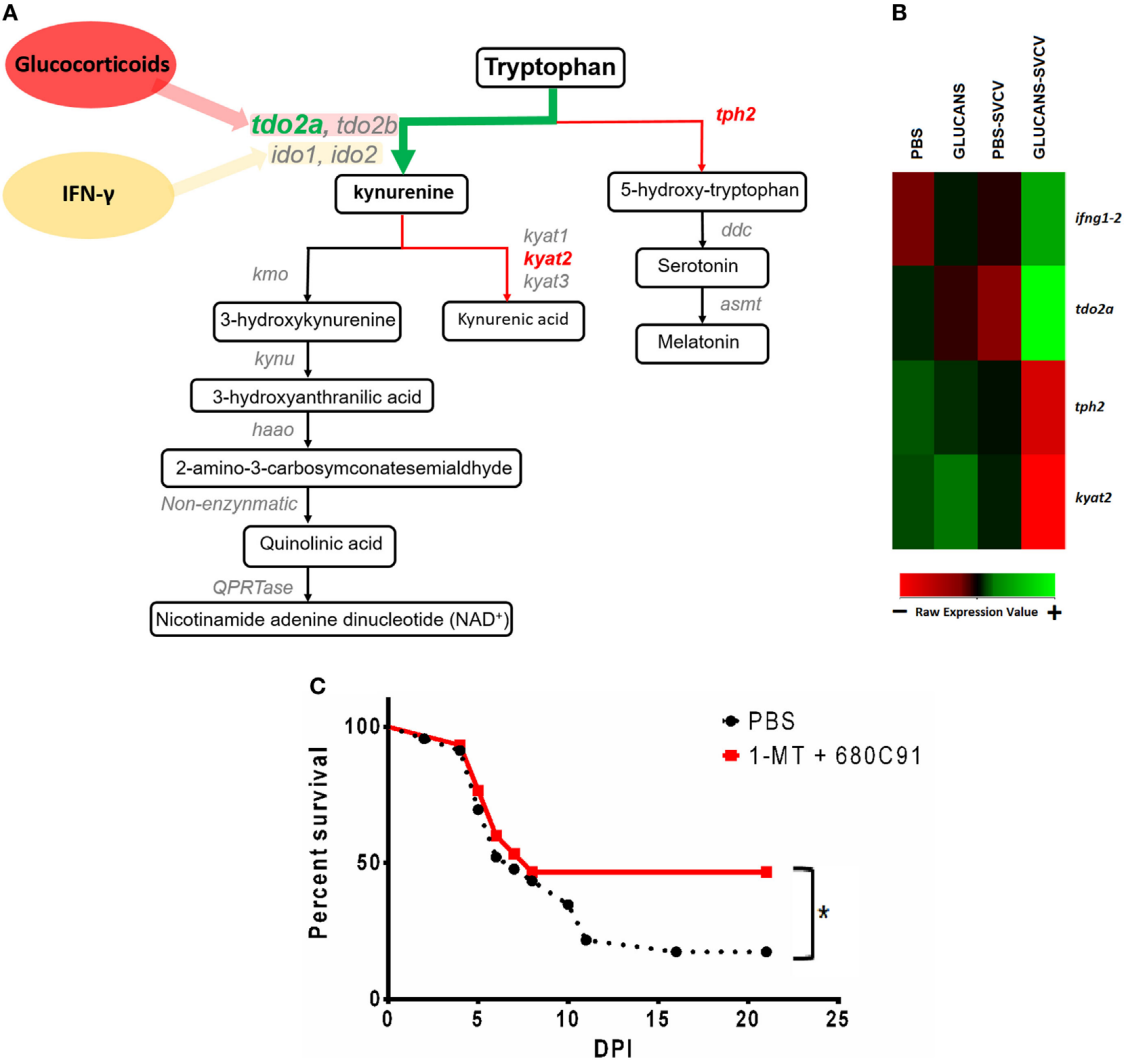
Analysis of the kynurenine pathway of tryptophan catabolism in zebrafish inoculated with β-glucans. **(A)** Schematic representation of the kynurenine pathway and the DEGs in the comparison Glucans-Spring Viremia Carp Virus (SVCV) vs. Glucans-PBS (*tdo2a, kyat2*) and the comparison Glucans-SVCV vs. PBS (*tph2*). **(B)** Heatmap representing some of the most affected genes after β-glucan treatment. Whereas *ifng1-2* and *tdo2a* are highly expressed in the Glucans-SVCV group, *tph2* and *kyat2* have lower expression levels in these fish. A color gradient scheme representing gene modulation (red: lower expression; green: higher expression) is shown on the bottom. **(C)** Kaplan–Meier survival curves representing the effect of a TDO-inhibitor (C80C91) and an IDO-inhibitor (1-MT) during a challenge with SVCV in adult zebrafish. A significant increase in the survival rate was observed in individuals inoculated with the inhibitors. Significant differences are represented by asterisks (*p < 0.05).

As observed in the GO enrichment analysis, the kynurenine pathway of tryptophan catabolism was highly altered in the Glucan-SVCV individuals compared to the PBS-SVCV fish (Figure 7A). Interestingly, this pathway has a close relation with the activity of IFN-γ. A heatmap including the genes that encode some of the main proteins involved in tryptophan catabolism, such as *ifng1-2*, tryptophan 2,3-dioxygenase a (*tdo2a*), tryptophan hydroxylase 2 (*tph2*), and kynurenine aminotransferase 2 (*kyat2*), revealed that previous stimulation with β-glucans determines the response of these genes to an SVCV challenge (Figure 7B). The genes *ifng1-2* and *tdo2a* were two of the most overexpressed genes in Glucans-SVCV vs. PBS-SVCV fish (Table 1). However, after short-term glucan stimulation, only *ifng1-2* was slightly overexpressed in individuals treated with glucans and then infected and *tdo2a* was not modulated (Figure 2 in Data Sheet S1 in Supplementary Material). Although it was not included in this heatmap, it is also interesting to highlight the second most upregulated gene in the Glucans group relative to the PBS group, the *G protein-coupled receptor 35 (gpr35)*, because intermediates of the kynurenine pathway are endogenous agonists of this receptor.

To investigate the importance of this pathway in mortality after SVCV, a TDO-inhibitor (C80C91) and an IDO-inhibitor (1-MT) were inoculated together with SVCV. The blockage of the kynurenine pathway with these inhibitors significantly increased the survival rate after infection (Figure 7C), with values that changed from 17.4% in untreated fish to 46.7% in individuals that received C80C91 + 1-MT. Therefore, the blockage of the kynurenine pathway significantly reduces the mortality caused by SVCV.

### Validation of the Microarray Data by qPCR

Correlation of the fold-change values for the comparison Glucans-SVCV vs. PBS-SVCV between microarray and qPCR showed a Pearson correlation coefficient (*r*) of 0.943 (*p* < 0.001). The expression data obtained for the selected genes are plotted in Figure 3 in Data Sheet S1 in Supplementary Material.

## Discussion

Typically, the use of immunostimulants, especially β-glucans, in aquaculture has been conducted with the objective of increasing the non-specific defense mechanisms of the cultured fish (2). Nevertheless, several factors, such as the timing and method of administration, must be carefully considered (2). An inappropriate treatment protocol can transform the immunostimulants into a double-edged sword for the teleost immune system, resulting in a stressful and immunosuppressive state. The long-term effects of immunostimulants, even after a single administration, can produce distress situation, with a negative impact on immune competence. Chronic stress involves metabolic changes that allow the organism to face the demand for resources that is associated with stress. A prolonged immune stimulus consumes energy resources due to the cost required to generate an appropriate immune response. When this demand is prolonged over time, these resources are exhausted, leading to immunosuppression (52).

The trained immunity induced by β-glucans seems to be limited to a period of approximately 20 days. This effect was observed after *in vitro* stimulation of murine spleen-derived monocytes with this substance (53) and after *in vivo* stimulation in zebrafish (32). Moreover, a work based on long-term protection against the virus IHNV after an i.p. injection of β-glucans in trout revealed that beyond 36 days poststimulation, a higher mortality rate occurred in pre-immunized individuals (28). The genes encoding proinflammatory cytokines, such as TNF-α, IL-1β, and IL-6, which are usually found to be upregulated during cell training (15, 32, 53) were not differentially modulated in our microarray results. These findings, together with the absence of protection against SVCV, led us to consider that in this work, we are describing long-term mechanisms that could persist beyond the end of training immunity.

In this work, we wanted to analyze the long-term effects of a punctual administration of three different PAMPs [LPS, poly(I:C), and β-glucans] on the immune system of zebrafish and their consequences during a viral infection. At 35 days posttreatment, none of the PAMPs was able to provide protection after infection, and higher mortalities were even observed for the three treatments, especially for β-glucans. Moreover, the transcriptome of fish that were previously treated with PAMPs was found to be highly altered, even after this long period of time, and this effect was more pronounced with β-glucans.

This is not the first time that a negative effect of β-glucans was observed in teleosts. The *in vitro* administration of β-glucans to turbot (*Scophthalmus maximus*) and gilthead seabream (*Sparus aurata*) phagocytes revealed that high doses of this molecule directly induced the respiratory burst and rapidly led to cell exhaustion (52), which may increase disease susceptibility. *In vivo* studies in large yellow croaker (*Larimichthys crocea*) showed that individuals fed high doses of β-glucans were less resistant to a challenge with *Vibrio harveyi*, probably because high doses resulted in immunosuppression or feedback regulation (54). A recent study in rainbow trout (*Oncorhynchus mykiss*) demonstrated that overdoses of β-glucans can lead to poor immune responses, probably due to the activation of stress response mechanisms (55).

The response triggered by a stressful stimulus is mediated by the release of stress hormones, such as glucocorticoids (GCs), which can increase or suppress certain pathways of the immune response depending on the intensity and duration of the stressor (36). While an acute stress is more closely associated with eustress (“good stress”), which involves short-term challenges that result in immunoenhancement, distress (“bad stress”) is caused by repeated or prolonged stress over time, which can cause immune suppression (56). In our case, in the short term (pre-stimulation with β-glucan and infection after 7 days), eustress could be responsible of the mechanisms that underlie training immunity and the slight protection observed after infection. In the long term (pre-stimulation with β-glucan and infection after 35 days), the distress generated by the high energy cost of prolonged training immunity could explain the lack of protection against SVCV and even the tendency toward an increase in mortality.

Several factors, mainly involved in cell metabolism, could explain the lack of protection against the viral challenge. In mammals, processes such as glycolysis, the pentose phosphate pathway, glutaminolysis, and cholesterol and fatty acid synthesis, are usually upregulated after β-glucan training, and the blockade of some of these pathways (such as glycolysis, glutaminolysis, and cholesterol synthesis) inhibits training immunity (57). The synthesis of lipids in immune cells is associated with cell proliferation and the generation of inflammatory cytokines; however, FAO is more closely associated with tolerance, the suppression of the immune response, memory, and a long cellular lifespan (58). The microarray revealed that genes related to fatty acid and cholesterol synthesis are inhibited in individuals treated with β-glucans, especially after viral infection, and genes related to FAO were overexpressed. In our results, *fasn, acc*, and *srebp1* mRNA levels decreased in immunostimulated fish. However, it is known that *mammalian target of rapamycin* (mTOR), the key player in training immunity activation (15), promotes the synthesis of fatty acids through the induction of these three genes (59). These results suggest a shift of training immunity to a more immunotolerant profile in our experiments.

Another remarkable aspect of the altered transcriptome of glucan-treated fish was amino acid depletion, which is another tolerance indicator. Among the most DEGs in the Glucans-SVCV group relative to the PBS-SVCV group are the TDO gene *tdo2a* and *homogentisate 1,2-dioxygenase* (*hgm*), which encode enzymes involved in amino acid catabolism. Phenylalanine and tryptophan are essential amino acids that are necessary for mTORC1 activation (60–62). TDO and indoleamine 2,3-dioxygenase (IDO), which catalyze the first step of tryptophan catabolism, are the rate-limiting enzymes of the kynurenine pathway (63). TDO is mainly produced in the liver but is expressed at immune-privileged sites and in some tumors and is associated with tolerance and immune evasion (17). Therefore, because tryptophan is degraded by the action of IDO and TDO in the kynurenine pathway, these enzymes act as inhibitors of mTOR activation. Indeed, the use of inhibitors of IDO activity, such as D-1MT, reversed the inhibitory effects generated by IDO on mTOR activation (64).

The intermediates of the kynurenine pathway, due to their toxic potential, may cause tissue damage and, as a consequence, generate higher stress, which can in turn interfere with the immune response to minimize tissue damage. It was recently observed that feed supplementation with kynurenic acid (KYNA) in rainbow trout had a toxic/stress-inducing effect, and this deleterious effect was related to the dose of KYNA (65). This pathological status was manifested during a subsequent infection with *Yersinia ruckeri*, with a higher mortality rate in animals that were administered a higher concentration of KYNA (65). On the other hand, kynurenic acid is an endogenous agonist of *gpr35* (which was the second most upregulated gene in β-glucan-stimulated zebrafish in the absence of infection), and its activation increases FAO and induces an anti-inflammatory state (66, 67). Additionally, kynurenic acid and kynurenine are endogenous agonists of the aryl hydrocarbon receptor (AhR) (68), which mediates immunosuppressive effects on the immune system (69), leading to inhibition of the promoter activity of IL-6 (70). Consistent with our results, the depletion of tryptophan from the medium impairs the antiviral response in mammals by inducing the formation of regulatory T cells and inhibiting the formation of effector T cells (71). These effects are observed in our transcriptome results, with a downregulation of the “T-helper 1 type immune response” and “positive regulation of interleukin-6 biosynthetic process” in the comparison Glucans-SVCV vs. PBS-SVCV. The regulation of the innate responses by the adaptive immune system is known in mammals (72) and also it has been reported in zebrafish (73).

The overexpression of the *ifng1-2* gene after viral infection in fish that previously received β-glucans initially contradicted the impaired response of these individuals. In fact, in our experiments, the overexpression of *ifng1-2* in zebrafish induced the activation of immune cells and was able to increase survival after an SVCV challenge. Nevertheless, a previous work reported an unexpected *in vivo* failure of Ifng1-2 to increase the resistance of zebrafish to bacterial and viral infections (74). IFN-γ is a pleiotropic cytokine, and it has been classically associated with a proinflammatory immune response (75). However, IFN-γ induces the expression of IDO, which catalyzes the same step as TDO in the kynurenine pathway (63). Therefore, the activation of the kynurenine pathway results from the induction of TDO through the action of GCs and from the induction of IDO through the overexpression of IFN-γ. This process not only depletes tryptophan from the medium but also probably interferes with the antiviral response of IFN-γ. The lack of induction of the *tdo2a* and the slight overexpression of *ifng1-2* compared to the infected controls in the short-term immunostimulation experiment would explain the increased survival of the individuals treated with glucans. Moreover, the higher viral resistance that was observed in this work when we applied the inhibitors of TDO and IDO together supports this hypothesis.

The depletion of tryptophan from the medium seems to be harmful for fish dealing with SVCV infection, as has been described for other viruses (76–78). However, it has been reported that toxic intermediates of the kynurenine pathway present bactericidal effects (76). Indeed, i.p. injection of β-glucans in *Salmo salar* was able to increase survival against Gram-negative bacteria, even after a long resting period (23). Interestingly, tryptophan depletion (by the action of TDO or IDO) and IFN-γ signaling can have opposite effects in the context of infection, depending on the type of pathogen (virus or bacteria) (76, 79).

In summary, long-term immunostimulation with β-glucans does not protect against SVCV infection. This pre-stimulation seems to lead the zebrafish immune system toward a strategy of immunosuppression and tolerization, which results in pernicious effects for responding to the virus. The interplay among GCs, lipid metabolism, the kynurenine pathway, IFN-γ, and mTOR activation seems to be the mechanism behind this response.

## Ethics Statement

Fish care and the challenge experiments were conducted according to the guidelines of the CSIC National Committee on Bioethics under approval number ES360570202001/16/FUN01/PAT.05/tipoE/BNG.

## Author Contributions

BN and AF conceived and designed the study. MA-R and PP performed the experimental procedures and data analyses. FR-L and LT conducted the microarray hybridizations. MA-R, PP, AF, and BN wrote the manuscript. All authors reviewed the manuscript.

## Conflict of Interest Statement

The authors declare that the research was conducted in the absence of any commercial or financial relationships that could be construed as a potential conflict of interest.
