# Supplementary material for: Analysis of the Long-Lived Responses Induced by Immunostimulants and Their Effects on a Viral Infection in Zebrafish (Danio rerio)
Source: Front Immunol. 2018 Jul 9;9:1575. doi: 10.3389/fimmu.2018.01575 (PMC6047052; doi:10.3389/fimmu.2018.01575)

Supplementary Material

Analysis of the long-lived responses induced by immunostimulants and their effects on a viral infection in zebrafish (*Danio rerio*)

Margarita Álvarez-Rodríguez, Patricia Pereiro, Felipe E. Reyes-López, Lluis Tort, Antonio Figueras, Beatriz Novoa*

*** Correspondence:** Beatriz Novoa: beatriznovoa@iim.csic.es

Supplementary Table 1

Primer sequences used in the *ifng1-2* expression plasmid (pcDNA3.1-*ifng1-2*) construction and qPCR analysis.

Gene	Ensembl ID	Use	Sense	Sequence (5'→3')
ifng1-2	ENSDARG00000024211	Plasmid expresion construction	F	ACTATGATTGCGCAACACATGATG
			R	TCAACCTCTATTTAGACTTTTGC
		qPCR	F	TTGTAGCTTCATCCACGCTG
			R	ATCTTCCTGCGAATCCTGAA
nkla	103909014	qPCR	F	GATGACGAATGACGGAGTAAAC
			R	TCTCATTCACAGCCCGGT
nkld	ENSDART00000064633	qPCR	F	TGTGATCAGATCGGGTTCCT
			R	AGCACAGATGGTTCTGGCAT
adm2a	ENSDARG00000045708	qPCR	F	TTGTCCAGGTGCATTGGAAG
			R	GTGTGGAGAGCAGGCTGATG
nr1d2a	ENSDARG00000003820	qPCR	F	CCGCATCTGAAACACTCAGC
			R	GTAAGCGAATGCACTGCTGC
mboat4	ENSDARG00000052110	qPCR	F	AAAGTGGAACCAAACCACGG
			R	CCACAGTGCAGAAAACCCAA
tdo2a	ENSDARG00000071429	qPCR	F	CGAGCGAAACATGCTCAAAG
			R	CCCCAATCTTGTTCTCCAGC
ccny	ENSDARG00000063677	qPCR	F	AAAGCAGCCAAGAAACGCTC
			R	GGGACACAGAAACCTCTGGC
pdk3a	ENSDARG00000014527	qPCR	F	TCCAGGCAGTTTATGTCCCC
			R	GGACAGATCCTCAATCCCCA
18s	ENSDART00000126982	qPCR	F	ACCACCCACAGAATCGAGAAA
			R	GCCTGCGGCTTAATTTGACT

Supplementary Table 2

Enrichment analysis of the DEGs in the comparisons Glucans vs. PBS, Glucans-SVCV vs. Glucans, PBS-SVCV vs. PBS and Glucans. BP: Biological process; MF: Molecular function.

**Glucans vs. PBS**			
**GO ID**	**GO Name**	**GO Category**	**P-Value**
GO:0045616	Regulation of keratinocyte differentiation	BP	0.006
GO:0014033	Neural crest cell differentiation	BP	0.002
GO:0007030	Golgi organization	BP	0.007
GO:0060536	Cartilage morphogenesis	BP	0.006
GO:0071871	Response to epinephrine	BP	0.006
GO:0030518	Intracellular steroid hormone receptor signaling pathway	BP	0.003
GO:0071788	Endoplasmic reticulum tubular network maintenance	BP	0.006
GO:0003881	CDP-diacylglycerol-inositol 3-phosphatidyltransferase activity	MF	0.006
GO:0004867	Serine-type endopeptidase inhibitor activity	MF	0.003
**Glucans-SVCV vs. Glucans**			
**GO ID**	**GO Name**	**GO Category**	**P-Value**
GO:0014033	Neural crest cell differentiation	BP	0.002
GO:0007030	Golgi organization	BP	0.007
GO:0060536	Cartilage morphogenesis	BP	0.006
GO:0030518	Intracellular steroid hormone receptor signaling pathway	BP	0.003
GO:0045616	Regulation of keratinocyte differentiation	BP	0.006
GO:0071871	Response to epinephrine	BP	0.006
GO:0071788	Endoplasmic reticulum tubular network maintenance	BP	0.006
GO:0004867	Serine-type endopeptidase inhibitor activity	MF	0.003
**PBS-SVCV vs. PBS**			
**GO ID**	**GO Name**	**GO Category**	**P-Value**
GO:0055114	Oxidation-reduction process	BP	0.005
GO:0001775	Cell activation	BP	0.009
GO:0051345	Positive regulation of hydrolase activity	BP	0.004
GO:0030301	Cholesterol transport	BP	0.006
GO:0070098	Chemokine-mediated signaling pathway	BP	0.005

GO:0070374	Positive regulation of ERK1 and ERK2 cascade	BP	0.005
GO:0030593	Neutrophil chemotaxis	BP	0.004
GO:0048247	Lymphocyte chemotaxis	BP	0.003
GO:0071356	Cellular response to tumor necrosis factor	BP	0.003
GO:0071347	Cellular response to interleukin-1	BP	0.003
GO:0071346	Cellular response to interferon-gamma	BP	0.003
GO:0002548	Monocyte chemotaxis	BP	0.002
GO:0007250	Activation of NF-kappaB-inducing kinase activity	BP	0.01
GO:0006067	Ethanol metabolic process	BP	0.01
GO:0042753	Positive regulation of circadian rhythm	BP	0.01
GO:0006642	Triglyceride mobilization	BP	0.005
GO:0002437	Inflammatory response to antigenic stimulus	BP	0.005
GO:0043153	Entrainment of circadian clock by photoperiod	BP	0.005
GO:0048020	CCR chemokine receptor binding	MF	0.002
GO:0032542	Sulfiredoxin activity	MF	0.005
GO:0005149	Interleukin-1 receptor binding	MF	0.005
GO:0031844	Type 4 neuropeptide Y receptor binding	MF	0.005
**Glucans-SVCV vs. PBS-SVCV**			
**GO ID**	**GO Name**	**GO Category**	**P-Value**
GO:0035023	Regulation of Rho protein signal transduction	BP	0.01
GO:0021779	Oligodendrocyte cell fate commitment	BP	0.007
GO:0007097	Nuclear migration	BP	0.006
GO:0002040	Sprouting angiogenesis	BP	0.003
GO:0045471	Response to ethanol	BP	0.006
GO:0002115	Store-operated calcium entry	BP	0.004
GO:0032288	Myelin assembly	BP	0.004
GO:0009620	Response to fungus	BP	0.006
GO:0006474	N-terminal protein amino acid acetylation	BP	0.002
GO:0072112	Glomerular visceral epithelial cell differentiation	BP	0.004
GO:0004198	Calcium-dependent cysteine-type endopeptidase activity	MF	0.002
GO:0004596	Peptide alpha-N-acetyltransferase activity	MF	0.001

Supplementary Figure 1

Expression of *ifng1-2* in 3 dpf zebrafish larvae microinjected in one-cell stage embryos with pcDNA3.1-*ifng1-2* or the empty plasmid (pcDNA3.1). The expression level of *ifng1-2* was normalized to the expression of the *18S ribosomal RNA* gene and expressed as the fold change with respect to the level detected in the control group (pcDNA3.1-injected). *ifng1-2* was overexpressed 74-fold in the individuals receiving the expression plasmid. The graph represents the means ± standard deviation of 3 independent biological replicates.

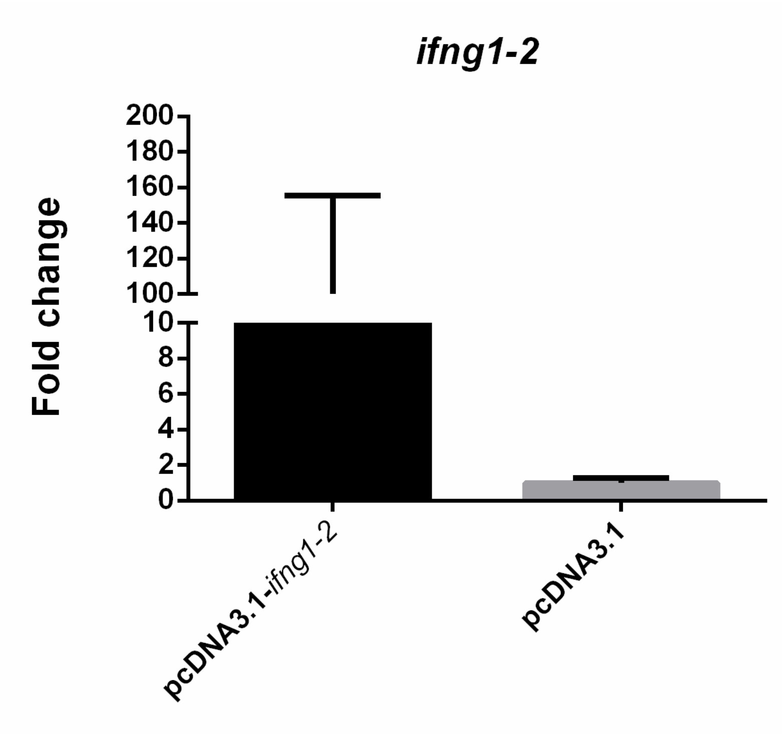

Supplementary Figure 2

Expression of *ifng 1-2* (**A**) and *tdo2a* (**B**) in adult zebrafish non-infected and infected after a rest period of 7 days of short-term priming with β-glucans. The expression level of the genes was normalized to the expression of the *18S ribosomal RNA* gene and expressed as the fold change with respect to the level detected in the control group (PBS). The graph represents the means ± standard deviation of 5 independent biological replicates.

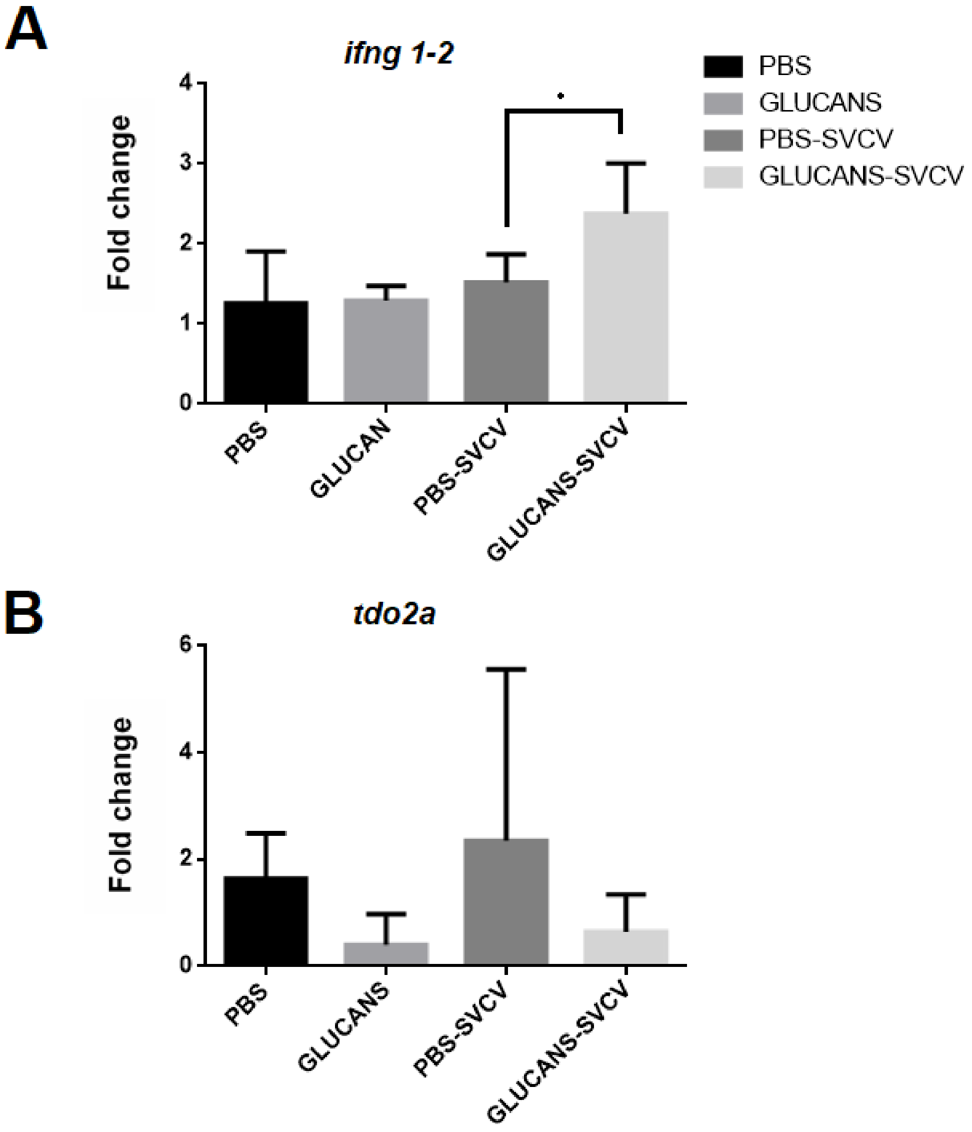

Supplementary Figure 3

qPCR validation of the microarray data. Correlation between microarray (x-axis) and qPCR (y-axis) data (Log10 fold-change) from 9 genes in the comparison Glucans-SVCV vs. PBS-SVCV. The correlation between microarray and qPCR data analyzed by the Pearson's correlation coefficient r = 0.943 and with a statistical significance of $p < 0.001$.

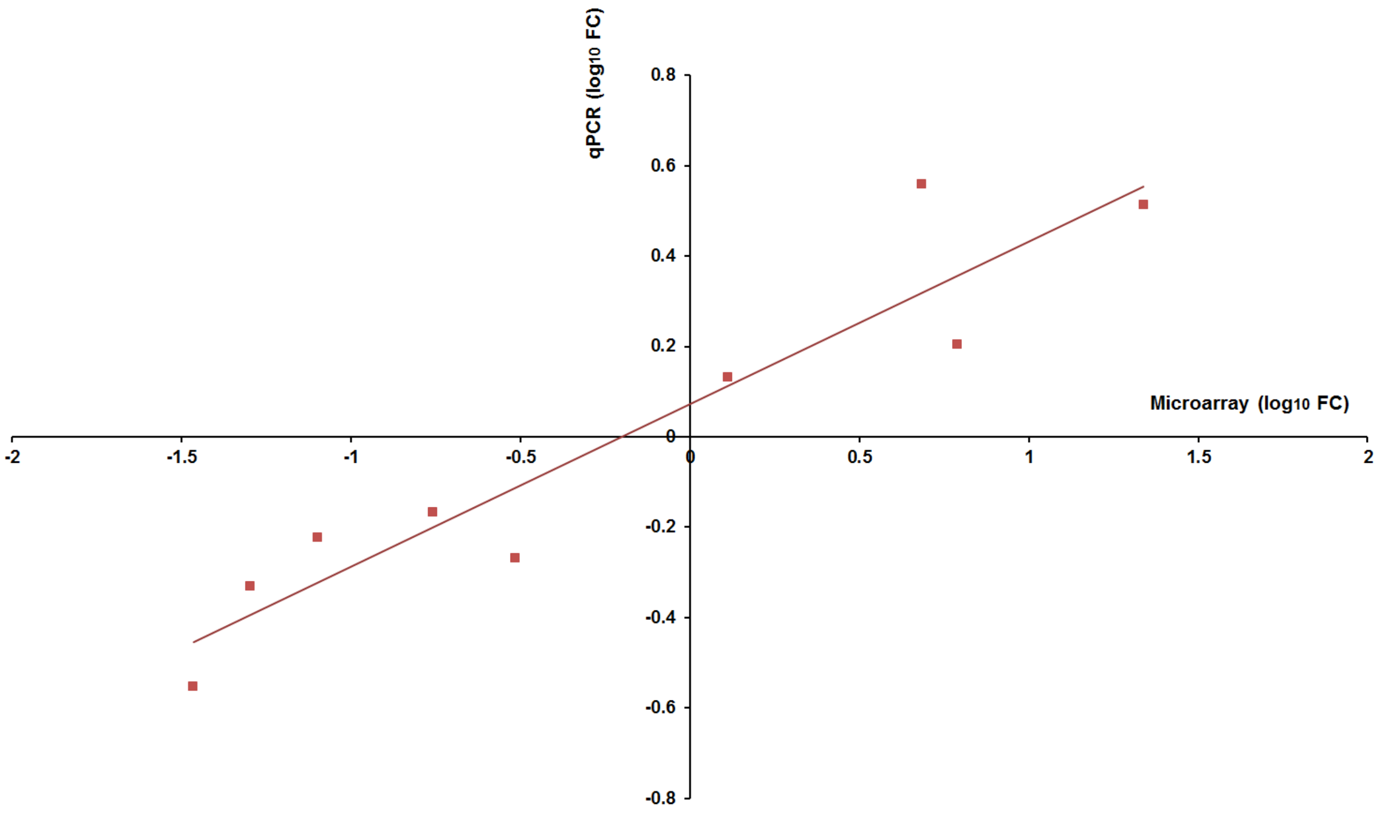

Supplement: Supplementary file 1 [file data_sheet_1.PDF]
